# Investigation of a Signal Demodulation Method based on Wavelet Transformation for OFDR to Enhance Its Distributed Sensing Performance

**DOI:** 10.3390/s19132850

**Published:** 2019-06-27

**Authors:** Kunpeng Feng, Jiwen Cui, Hong Dang, Xun Sun, Dong Jiang, Yihua Jin, Yizhao Niu, Xuping Zhang

**Affiliations:** 1 Institute of Optical Communication Engineering, College of Engineering and Applied Sciences, Nanjing University, Nanjing 210093, China; 2 Key Laboratory of Intelligent Optical Sensing and Manipulation, Ministry of Education, Nanjing 210093, China; 3 Center of Ultra-precision Optoelectronic Instrument, Harbin Institute of Technology, Harbin 150080, China; 4 Key Lab of Ultra-precision Intelligent Instrumentation (Harbin Institute of Technology), Ministry of Industry and Information Technology, Harbin 150080, China

**Keywords:** Optical distributed sensing, wavelet transforms, OFDR

## Abstract

Optical fiber distributed sensing that is based on optical frequency domain reflectometer (OFDR) is a promising technology for achieving a highest spatial resolution downwards to several millimeters. An OFDR signal demodulation method that is based on Morlet wavelet transformation (WT) is demonstrated in detail to improve the resolution of distributed sensing physical quantity under a high spatial resolution, aiming at the trade-off between spatial and spectrum resolution. The spectrum resolution, spatial interval of the measured gauges, and spatial resolution can be manually controlled by adjusting the wavelet parameters. The experimental results that were achieved by the wavelet transformation (WT) method are compared with these by short time Fourier transformation (STFT) method and they indicate that significant improvements, such as strain resolution of 1 με, spatial resolution of 5 mm, average repeatability of 4.3 με, and stability of 7.3 με within one hour, have been achieved. The advantages of this method are high spatial and spectral resolution, robust, and applicability with current OFDR systems.

## 1. Introduction

Optical fiber distributed sensors (OFDS) show high potential in the fields of structural health monitoring, radiation detecting, reconstruction of three-dimensional (3D) shape, and pipeline surveillance, for their capability of carrying high dense information along a single fiber, being immune to electromagnetic interference and high spatial and spectrum resolution [1,2,3]. OFDS have completed the transition from quasi-distributed to real-distributed sensing over the past 20 years with the development of the intrinsic backscattering light in optical fiber [1]. Among the OFDS of different measuring principles, optical frequency domain reflectometer (OFDR) that is based on intrinsic Rayleigh scattering (RS) has been given tremendous attention as a result of its high spatial and spectrum resolution and without sensing the dead-zone.

Each segment within an optical fiber contains a range of spatial points with random and “frozen” RS, which can be modeled as a very weak fiber Bragg grating (FBG) with a random period. The external variation of strain/temperature would lead to the scattering spectrum shifts of these segments. OFDR is used to interrogate the wavelength shifts of RS spectrum, and the distributed sensing along a common single mode fiber can thus be achieved. Lots of achievements have also been made in the demodulation methods of OFDR system [4,5,6]. A short time Fourier transformation (STFT) based method is utilized in a conventional method to demodulate the external distributed sensing information (strain, temperature, etc.). This method utilizes fast Fourier transformation (FFT) to process the interference signal of OFDR and obtain the distributed reflection amplitude along the fiber under test (FUT) in the spatial domain. After that, a sliding window along the FUT extracts distributed information at corresponding positions and inverse fast Fourier transformation (IFFT) is employed to achieve the RS spectrum in the wavelength domain. Wherein, the bandwidth (number of data points) and location of the sliding window, respectively, determine the spatial resolution and the current analyzing position. Subsequently, the peak shift of the cross-correlation between reference and measurement RS spectrum could respect the distributed sensing information along the FUT. An increment of the data number of the RS spectrum will improve the uncertainty of the wavelength shift during the IFFT of signals from spatial domain into wavelength domain, but at a cost of the spatial resolution. It can be clearly concluded that it is a trade-off between the spectrum resolution and spatial resolution in the conventional STFT based method [7]. Several new techniques, such as linear optical sampling and time-stretch method, were proposed to simultaneously improve the spatial and spectrum resolution [8,9]. However, their valid sensing length is only several centimeters, and these techniques cannot satisfy the requirement of distributed sensing. A new OFDR demodulation method for high performance OFDS is highly demanded.

The demodulation of OFDR signals to achieve distributed sensing information can be identified as a time-frequency analysis process. The disadvantages of the STFT, such as a trade-off between time and frequency resolution and unadjustable time-frequency analysis window, can be avoided by employing a wavelet transformation (WT) time-frequency tool. Therefore, an OFDR demodulation method that is based on WT is proposed to solve the trade-off problem between the spectrum resolution and spatial resolution, and the spectrum resolution can be significantly improved, especially under a high spatial resolution. This paper is organized, as follows. Section 2 demonstrates the relationship between the distributed sensing parameters and wavelet parameters, and how to adapt Morlet WT to demodulate the distributed sensing information of OFDR. The experimental setups and performance tests of the OFDR demodulation method based on WT are shown in Section 3. The conclusion of this paper is given in Section 4.

## 2. Principle of the OFDR Demodulation Method Based on WT

In this section, the basic principle of OFDR is introduced, and the relationship between the spectrum (time) and position (frequency) is established. Subsequently, WT time-frequency analysis method and how to adapt WT to demodulate distributed sensing information are demonstrated. Finally, the influences of the WT’s parameters on distributed sensing are discussed and problem during the application of WT in demodulating OFDR signal is solved.

### 2.1. Basic Principle of OFDR

**Figure 1:**
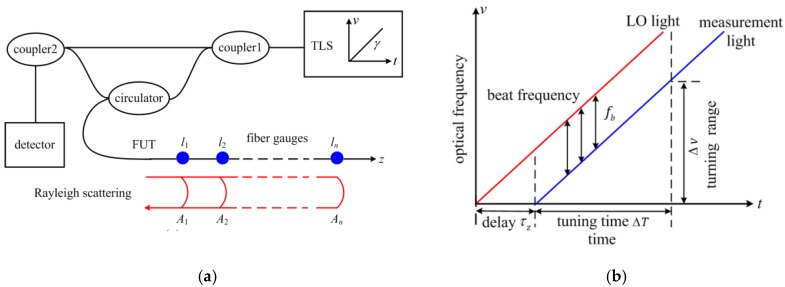
Basic principle of optical frequency domain reflectometer (OFDR). (**a**) schematic diagram of a basic OFDR configuration. (**b**) interference between local oscillator (LO) and measurement light.

Figure 1a illustrates a basic OFDR configuration. It consists of a tunable laser source (TLS), two optical fiber couplers, a circulator, and FUT for distributed sensing. The main structure of OFDR is an interferometer for local oscillator (LO) light from TLS and measurement light (distributed reflected light) from FUT. Weak FBGs or RS in FUT can influence the distributed reflected light from FUT. On the other hand, the spectrum of the local fiber gauge varies with the subjected strain/temperature whether the fiber gauge is FBG or a common single mode fiber. Accordingly, the reflected light from FUT carries the distributed sensing information.

The spectrum of fiber gauges that are located at different positions along the fiber can be differentiated by delay time. The TLS performs linear-frequency-sweeping and transforms the delay times into beat frequency variations in the OFDR interferometer during the distributed sensing. The normalized signal of a Dirac fiber gauge received by a DC-blocked detector can be written as:

(1) $$\tilde{I}(t,z)={\tilde{R}}_{z}(\gamma_{0}+\gamma_{v}t)\cos\left\{2\pi \left[v_{0}\tau_{z}+\gamma_{v}\tau_{z}t+\frac{1}{2}\gamma_{v}\tau_{z}{}^{2}\right]\right\}$$

where, $v_{0}$ is the initial frequency of the TLS, $\gamma_{v}$ is the linear-frequency-sweeping velocity, $\tau_{z}$ is the delay time between LO and measurement light, and ${\tilde{R}}_{z}(t)$ is the normalized reflectivity or the spectrum of the Dirac fiber gauge.

Figure 1b indicates that the beat frequency of the interference signal is proportional to the delay time:

(2) $$f_{b}=\gamma_{v}\tau_{z}=\frac{2nz\gamma_{v}}{c}$$

where, *z* is the length from the position of zero delay time to the Dirac fiber gauge and *n* is the refractive index of the FUT.

The reflectivity or the spectrum of the Dirac fiber gauge can be achieved through FFT and IFFT at a certain beat frequency. Fiber gauges of different locations can be differentiated by their frequencies. Distributed sensing can be accomplished based on Equations (1) and (2). However, RS is continuous along the fiber and there are lots of fiber gauges in FUT. The normalized signal is an integral of every fiber gauges within the whole frequency-sweeping time:

(3) $${\tilde{I}}_{\mathrm{FUT}}(t)=\int_{0}^{L}\tilde{I}(t,z)dz$$

where, *L* is the length of FUT.

It can be seen from Equation (3) that the OFDR signal is a combination of signals of different frequencies and the location of the fiber gauge is proportional to the beat frequency. A swept laser is utilized based on the principle of OFDR and the sampled time is proportional to the wavelength or optical frequency. The measured signal of an OFDR system in time-domain can be related to the wavelength of the swept laser source:

(4) $$\lambda (t)=\lambda_{0}+\gamma t$$

where, $\lambda_{0}$ is the initial wavelength of the swept laser source and γ is the swept velocity.

At the moment of $t_{0}$, the measured signal of an OFDR system reflects the sensing information of the wavelength $\lambda (t_{0})$. On the other hand, Equation (2) indicates that the reflected position is related to the beat frequency of the measured signal. One can achieve the distributed sensing data by using a time-frequency analysis method at the moment of $t_{0}$, wherein the *x* and *y* axes, respectively, represent position along the sensing fiber and reflectivity of the wavelength $\lambda (t_{0})$.

**Figure 2:**
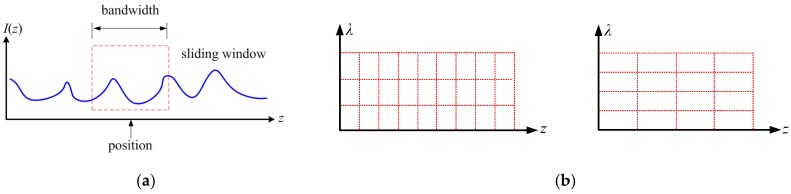
Demodulation of OFDR signal to achieve the distributed sensing information: (**a**) sliding windows in the spatial domain; (**b**) comparison of short time Fourier transformation’s (STFT’s) spatial and spectrum resolution, left has better spatial resolution and right has better wavelength resolution.

Therefore, Figure 2a illustrates the conventional OFDR demodulation process. A sliding window is adapted to the OFDR signal in the position-domain that goes through FFT of original OFDR signal. Based on Equation (1), the signal component within the frequency window is related to the spectrum information of the fiber gauge. On the other hand, the position and bandwidth of the window, respectively, respect to the position along the FUT and spatial resolution of the distributed sensing. The point number within the sliding window or the spatial resolution determines the spectrum resolution. Accordingly, it is a trade-off between spatial and spectrum resolution. Subsequently, IFFT is applied to the signal component within the window and the spectrum of the corresponding fiber gauge is thus obtained. This process can be equivalent to a STFT spatial-spectrum analysis.

A high frequency signal should be analyzed using a narrow window to achieve a high time resolution based on the basic principle of time-frequency analysis; a low frequency signal should be analyzed while using a broad window to achieve a high frequency resolution. Figure 2b indicates the comparison of STFT’s spatial and spectrum resolution: left has better spatial resolution and right has better wavelength resolution. Figure 2b shows that the time-frequency analysis window of STFT is interdependent, which describes the trade-off between spatial and spectrum resolution in the OFDR demodulation method that is based on STFT. It also indicates that the frequency-time window of STFT is fixed and it cannot be automatically adapted to the frequency. In the distributed optical fiber sensing, for the fiber gauges near the beginning of FUT (beat frequency is lower), a wide window should be applied and the spatial resolution is relatively lower. However, a narrow window should be utilized for the fiber gauges of a long distance and the spatial resolution can be higher.

### 2.2. OFDR Demodulation Method Based on WT

Based on the principle of OFDR, it can be identified as an optical time-frequency signal analysis problem, and the spectrum and location of OFDR are, respectively, corresponding to time and frequency domain. The combination of the WT and OFDR would improve the distributed sensing performance of OFDR. The trade-off problem between spectrum and spatial resolution can be solved and the spectrum resolution is significantly enhanced.

The discrete WT (DWT) of the measuring signal can be written as [10,11]:

(5) $$W(a,b)=\sum_{n=1}^{N}{\left|a\right|}^{-1/2}w(n)\varphi^{*}(\frac{n-b}{a})$$

where, W(a,b) is the wavelet coefficient that represents the energy in the corresponding frequency range, w(n) is the measured time-domain signal of OFDR, φ(n) is the mother wavelet, a and b is the scaling factor and translation factor, $\varphi^{*}$ represents the complex conjugate of φ(n), and *N* is the data length of the sample OFDR signal.

**Figure 3:**
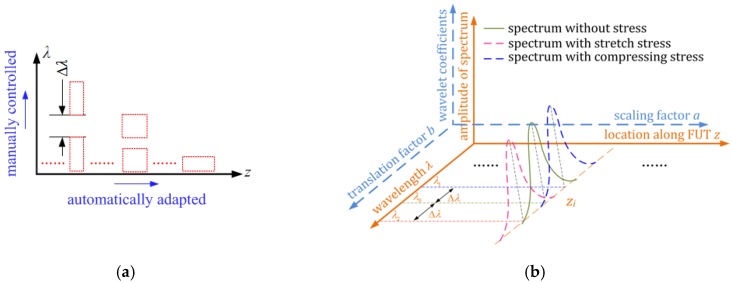
Diagram of demodulating OFDR signals by wavelet transformation (WT) method. (**a**) the spectrum-position characteristics of WT; (**b**) the relationship between the distributed sensing and WT parameters.

The intrinsic limitation of STFT on time-frequency analysis leads to a rapid decrease of spectrum resolution with an increment of spatial resolution and their trade-off is very significant. However, the WT tool can always keep the uncertainty principle during the time-frequency analysis process. The time and frequency resolution can be automatically adapted. As shown in Figure 3a, axis *x* is in the frequency domain and it represents spatial location *z* long the fiber; axis *y* is in time domain and represents the wavelength. WT has a better time resolution for signal of a high frequency. However, frequency resolution is paid more attention for signal of a low frequency rather than a high time resolution. Hence, the time window of WT method is automatically varied with the frequency and it keeps an appropriate frequency resolution. The most difference between WT and STFT is the time-frequency transformation theory: WT uses the instantaneous base signal of different frequencies in time domain to analyze the time-frequency characteristics of signal. The time resolution would get worse with the increment of analyzed frequency due to the automatic time-frequency adaptation of WT. However, the instantaneous base signal has a narrow bandwidth in frequency-domain and a short duration in time-domain. The bandwidth of the instantaneous base signal in spectrum-domain is would be much narrower than that of STFT method and about several pm when using the WT method. The spatial and spectrum resolution vary with the location based on the characteristics of the WT method. Generating new scaling factors in different divided segments can solve the location-varying spatial resolution, which will be discussed in the following. In every divided segment, the location-varying spectrum resolution can be ignored, because its bandwidth in spectrum-domain is several pm and the spectrum resolution is manually controlled by the translation factors. Accordingly, the time and frequency resolution can be independently controlled, and this method can reduce the trade-off between time and frequency resolution. While, it is fixed when the frequency resolution is once determined in the STFT method. By this characteristic of WT, the spectrum or measured physical quantity resolution can be manually selected of different locations.

The WT base can be built according to the time-frequency analysis requirements. WT could achieve a better time resolving performance, which the frequency resolution hardly influences, and information in the time-domain is thus much more abundant. This method is significantly different from the STFT method that is based on padding zeros, which gets more information by interpolation. However, WT can directly and accurately achieve frequency-domain characteristics through instantaneous WT bases of different frequencies in time-domain.

In the WT method, the frequency and observed time can be controlled by the scaling and translation factors. Hence, the wavelet series can be thus regarded as a series of local time-frequency transformation windows with varying frequency:

(6) $$\left\{ \begin{array}{l} a_{i}=f_{c}/f_{i}\\ b_{i}=t_{i} \end{array}\right.$$

where, $a_{i}$ and $b_{i}$ is the scaling series and translation series, $f_{c}$ and $f_{i}$ is the central frequency of the mother wavelet and wavelet series, and $t_{i}$ is the location of the observed time windows.

The amplitudes of the different frequency elements can be extracted by a series of bandpass filters (BPF) that were formed by wavelet series with different scaling factors. The amplitudes at different observed times can be extracted by a series of time windows that formed by wavelet series with different translation factors. In Figure 3b, a series of BPF with different central frequencies and different observed times is achieved and the time-frequency characteristics of the measured signal can be thus analyzed.

In an OFDR system, an auxiliary interferometer generates an equal-wavelength-sampling clock. The beat frequencies of the measurement interferometer are proportional to the position of the measured gauges along FUT and the time-varying amplitude of a certain frequency element is related to the RS spectrum of the corresponding measured gauge. By using WT in the processing of OFDR signals, a series of BPF of different central frequencies can extract the distributed sensing information of different measured gauges and the time-varying location of the BPF is related to the wavelength. The two-dimensional (2D) scaling and translation series in time-frequency WT is thus equivalent to a 2D location-wavelength series in OFDR, as shown in Figure 3b. The wavelet coefficients represent the amplitudes of the RS spectrum in spatial-spectrum domain. Accordingly, the relationship between wavelet factors and the distributed sensing can be written as:

(7) $$\left\{ \begin{array}{l} a_{i}=cf_{c}/2nz_{i}\gamma \\ b_{i}=\left(\lambda_{i}-\lambda_{0}\right)/\gamma \end{array}\right.$$

where, $z_{i}$ is the location of the measured gauge, $\lambda_{i}$ is the wavelength of the current wavelet, n is the refractive index of FUT, γ is the swept velocity of the tunable laser source, c is the velocity of light, and $\lambda_{0}$ is the initial swept wavelength.

Based on the Equation (7), the wavelet coefficients in a 2D space (*a_i_*, *b_i_*) are related to the amplitudes of the distributed reflection spectrum in a 2D space (*z_i_*, *λ_i_*), as illustrated Figure 3b. It also shows that the spectrum shift of a certain gauge is proportional to the shift of translation factor and cross-correlation calculation can be utilized to achieve the spectrum shift. One can achieve the WT coefficient (*z_i_*, *λ_i_*) by WT of OFDR signal with the proper scaling and translation series. The WT coefficient (*z*_0_, *λ_i_*) represents the RS spectrum of a sensing gauge located at position *z*_0_. Cross-correlation of the reference and measurement RS spectrum is then calculated to achieve a spectrum shift that is proportion to strain or temperature variation. Cross-correlation is successively calculated to get distributed sensing data. Accordingly, a new signal processing method for demodulating the distributed sensing information of OFDR is thus proposed.

Subsequently, the mother wavelets and how it influences the distributed sensing are investigated. The time-frequency transformation results are highly dependent on the characteristics of the mother wavelet. In general, a mother wavelet of a high form similarity with the measured signal is chosen in the application. Morlet wavelet, which is usually used in the fields of seismic wave, voice, heartbeat, and some linear frequency modulating signal analysis, is a complex-sine-modulated Gauss wavelet. In this paper, Morlet wavelet is employed as the mother wavelet in the distributed sensing. The Morlet wavelet can be expressed in a discrete form as:

(8) $$\varphi (n)=\frac{1}{\sqrt{\sigma \pi }}e^{j\omega_{0}n}e^{-n^{2}/\sigma }$$

where, σ and $\omega_{0}$ are related to the bandwidth and central frequency of the mother wavelet.

The central frequency of the mother wavelet can be related to the spatial position of distributed sensing, when a=1:

(9) $$\omega_{0}=2\pi f_{c}=4\pi nz_{c}\gamma /c$$

The bandwidth of the Morlet wavelet of different scale factors in frequency can be written as:

(10) $$\Delta f=\frac{1}{2\pi a_{i}\sqrt{\sigma }}$$

The bandwidth of the mother wavelet is related to the spatial resolution. Based on Equations (2), (6), and (7), the bandwidth of the Morlet wavelet of different scale factors can be written as:

(11) $$\Delta f_{i}=\frac{n\gamma \delta X_{i}}{\pi c\sqrt{\sigma }}$$

where, $\delta X_{i}$ is the window bandwidth in the location space.

Besides, the relationship between window bandwidth in the frequency and location space can be expressed as:

(12) $$\Delta f_{i}=\frac{2n\gamma \delta X_{i}}{c}$$

Hence, the relationship between the window bandwidth in the location space (representing spatial resolution) and the bandwidth of the mother wavelet can be achieved:

(13) $$\delta X_{i}=\frac{c}{4\pi n\gamma \sqrt{\sigma }}\frac{z_{i}}{z_{c}}$$

Equation (13) demonstrates that the window bandwidth in the location space is determined by σ, which should be set according to the demand spatial resolution.

Based on Equation (13), the spatial resolution varies with location in the WT method. Equation (9) also indicates that the spatial or frequency bandwidth can be controlled by scale factor. Accordingly, the analysis signal is divided into several segments. The central frequency of each segment is different and the spatial or frequency bandwidth can be kept in an acceptable range. The segments can be determined in the following method:

(14) $$\left\{ \begin{array}{l} z_{i}=K_{\delta X}z_{i-1}(i=2,3,\cdots )\\ K_{\delta X}=\delta X_{\max}/\delta X_{\mathrm{set}} \end{array}\right.$$

where, $z_{0}$ is initial distributed sensing location, $\delta X_{\max}$, $K_{\delta X}$, and $\delta X_{\mathrm{set}}$ are, respectively, the maximum variation spatial resolution, the maximum variation ratio, and the set spatial resolution.

**Figure 4:**
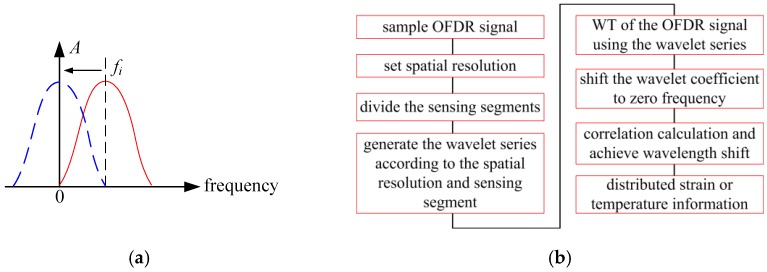
OFDR demodulation method based on WT. (**a**) the diagram of the wavelet equivalent BPF. (**b**) The signal processing flow of the proposed OFDR demodulation method.

Each wavelet can be considered as a BPF in the frequency or location domain. Therefore, the signal of wavelet coefficients is located in intermediate frequency (IF) and it contains IF noise. Figure 4a illustrates the diagram of the wavelet equivalent BPF and its central frequency is $0.5\Delta f_{i}$. The RS spectrum should be shift by a frequency of $0.5\Delta f_{i}$ to zero frequency and the IF noise would be filtered to reduce the IF noise. This method can filter the IF noise and the curves are smoother. Besides, the cross-correlation result only has one peak and the wavelength shift can be clearly extracted.

In conclusion, an OFDR demodulation method that was based on WT is demonstrated as above and Figure 4b illustrates the signal processing flow:

(1) Sample OFDR signal.
(2) Set the spatial based on the requirement. In Equation (13), $\delta X_{i}$ is the spatial resolution when $z_{i}=z_{c}$. Hence, σ can be determined with an acknowledgement of an OFDR system.
(3) Divide the sensing segments while using the method demonstrated in Equation (14). One can determine the length of a segment with the initial position of segments once the acceptable spatial resolution variation is confirmed. Accordingly, the segments are divided in this way.
(4) Generate the wavelet series of each sensing segments according to the spatial resolution. Based on Equation (7), the scaling and translation factors are generated, and their interval are, respectively, spatial and wavelength interval. At the same time, the wavelet base function can be achieved while using Equation (8).
(5) WT of the OFDR signal with the wavelet series. One can do WT of the OFDR signal with the wavelet series that are generated in step (4) while using Equation (5).
(6) Shift RS spectrum to zero frequency to reduce the IF noise. Multiply the RS spectrum with a cosine IF signal and then put the signal through a lowpass filter.
(7) Cross-correlation calculation of the reference and measurement RS spectrum and achieve the wavelength shift of each sensing gauge.
(8) Reconstruct the distributed strain or temperature information according to their sensitivity verse wavelength shift.

## 3. Experiments on Distributed Sensing Performance

In this section, the OFDR distributed sensing performances are experimentally tested and compared with the conventional demodulation method that is based on STFT.

**Figure 5:**
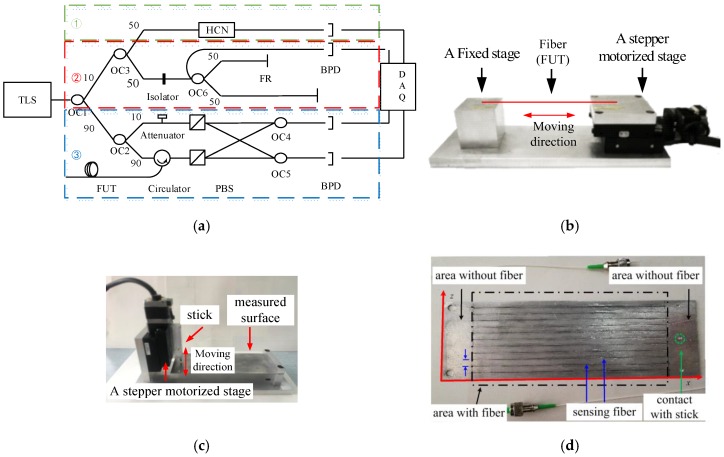
Schematic of the experimental systems: (**a**) configuration of the OFDR distributed sensing system, wherein OC is optical coupler, FR is Faraday mirror, BPD is balanced photodetector, PBS is polarization beam splitter, HCN is hydrogen cyanide gas cell, and DAQ is data acquisition card; (**b**) experimental setups of stretching fiber device for generating distributed strain; (**c**) experimental setups of surface strain testing device; and, (**d**) the fiber glued on the Aluminum alloy plain.

Figure 5a illustrates the experimental setups of the OFDR distributed sensing system [12,13]. The data acquisition (DAQ) card is PCI-1714 (Advantech, TaiBei, China). The balanced photodetector (BPD) is a polarization dependent balanced detector INT-POL-1550 (Thorlabs, Newton, USA) that is produced by Thorlabs. The external cavity tunable laser source is T100R produced by Yenista. The HCN gas cell is H13C14N produced by Wavelength Reference, which is equivalent of the NIST SRM 2519. The other optical fiber devices are all produced by Advance Fiber Resources. Figure 5b,c show the devices for stretching fiber and testing surface strain, which can generate controllable distributed strain. A stepper motorized stage Suruga Seiki KXC06 (Suruga Seiki, Shizuoka, Japan) is employed to generate micro displacements downwards to 50 nm. In Figure 5b, the fiber under test (FUT) is a section of fiber sensing between 8.2 m to 8.7 m, and it is glued on the stepper motorized stage and a fixed stage. The FUT is glued on the surface of an Aluminum alloy cantilever for sensing surface strain, as in Figure 5c. Figure 5d shows that fiber sensing is glued like “snake” on the surface in the area of 20–140 mm along axis *x* and 5–55 mm along axis *z*. The length, width, and thick of the Aluminum alloy plain are, respectively, 160 mm, 60 mm, and 1 mm. Its Young’s module is ~69 GPa. The length of the sensing segments along axis *x* is 120 mm and their interval along axis *z* is 5 mm.

### 3.1. Experiment on the OFDR Demodulation Method Based on WT

**Figure 6:**
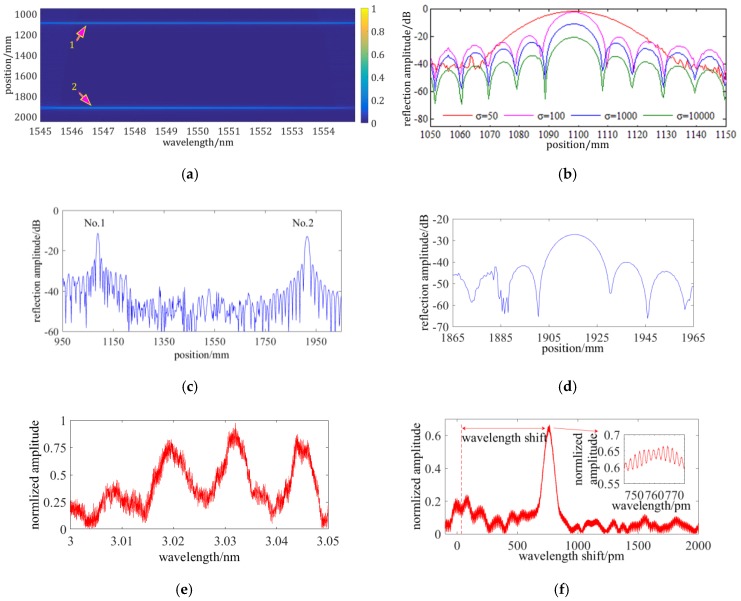

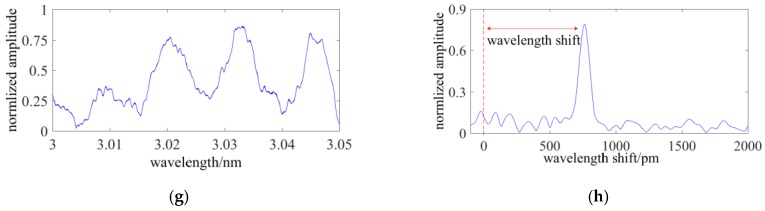
Experimental results of OFDR based on WT method: (**a**) distributed sensing result of two FC/APC reflective end surfaces; (**b**) the influence of the σ on the spatial resolution; (**c**) the spatial resolution of first and second FC/APC reflective end surfaces; (**d**) the distributed reflection spectrum of the No.2 FC/APC reflective end surface processing by the method demonstrated in Equation (13); (**e**) Rayleigh scattering (RS) spectrum of a fiber sensing gauge located at 1.4 m without frequency shift, the wavelength of x axis is relative to 1550 nm; (**f**) cross-correlation result of RS spectrum without frequency shift; (**g**) RS spectrum of a fiber sensing gauge located at 1.4 m with frequency shift, the wavelength of x axis is relative to 1550 nm; and, (**h**) cross-correlation result of RS spectrum with frequency shift.

An OFDR signal combining two FC/APC reflective end surfaces is processed while using WT method. The laser sweep range is from 1545 nm to 1555 nm and sweep rate is ~100 nm/s. Figure 6a shows the demodulation result. Two FC/APC end surfaces are respectively located at ~1098 mm and ~1915 mm, and there are two high reflection peaks at these locations. The No.1 FC/APC end surface located at~1098 mm is taken as an example to investigate the influence of the mother wavelet parameters on the spatial resolution.

Figure 6b shows that the 3 dB spatial width of the reflective surface is, respectively, 25 mm, 10 mm, 8 mm, and 5 mm when the bandwidth control parameter *σ* is 50, 100, 1000, and 10000. However, the descent rate of the spatial width gets lower with increasing the value of σ. The spatial resolving limitation is close to 5 mm and it cannot be further improved by increasing the value of *σ*. The frequency resolving performance of the WT method mainly causes the limitation. At the same time, the dynamic range gets lower with the decrease of the spatial resolution as a result of that a BPF of a narrower bandwidth is utilized and the signal intensity passing this BPF gets weaker.

Figure 6c shows that the bandwidth of the No.1 FC/APC end surface in location space is ~5 mm. However, that of the No.2 is ~7 mm under a same condition. This is caused by that the window bandwidth in the location space is varying with the location. To reduce the influence of the location-varying spatial resolution, a maximum variation ratio threshold is set and the wavelet series is re-generated when the spatial resolution variation ratio is over the threshold. Accordingly, the FUT is divided into several segments and a series of wavelet of different σ is generated to demodulate the distributed sensing information. The distributed sensing location is set from 950 mm to 1965 mm and the $K_{\delta X}$ is 10%. The segments are divided using the method demonstrated by Equation (13) to generate a new wavelet series [*a_i_*, *b_i_*]. By this way, the No.2 FC/APC end surface is located in the eighth segment. Its distributed reflection spectrum is illustrated in Figure 6d and the bandwidth is reduced from ~7 mm to ~5.3 mm. Hence, the degeneration of spatial resolution can be reduced into a controllable range by different divided segments. The spatial resolution is automatically adapted in each segment.

A fiber sensing gauge located at 1.4 m is measured and it is processed by the WT method. The laser sweep range is from 1545 nm to 1549 nm and sweep rate is ~100 nm/s. As the analysis above, the signal of wavelet coefficients or the RS spectrum containing IF noise is shown in Figure 6e. Cross-correlation calculation is conducted to achieve the wavelength shift and Figure 6f shows the result. The zoom-in illustration in Figure 6e shows that the cross-correlation peak has multi fluctuant peaks that are caused by the IF noise of the RS spectrum. To reduce the IF noise, the RS spectrum should be shift by a frequency of $0.5\Delta f_{i}$ to zero frequency and the IF noise would be filtered. After frequency shift, the RS spectrum and the cross-correlation result are, respectively, shown in Figure 6g,h. This method can filter the IF noise and the curves are smoother. Besides, the cross-correlation result has only one peak and the wavelength shift can be clearly extracted.

### 3.2. Experiment on the Sensing Nonlinearity

**Figure 7:**
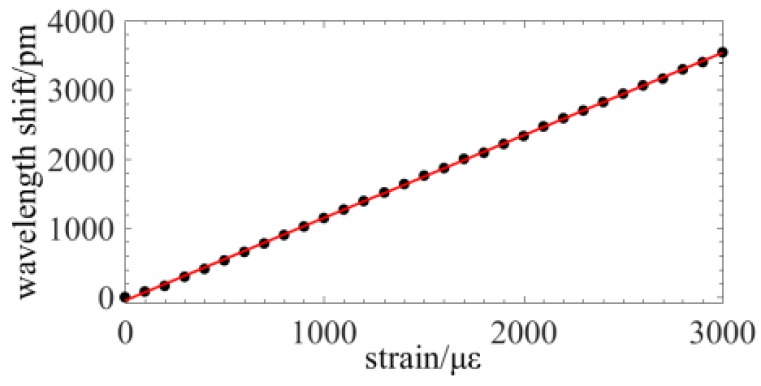
Experiment on the sensing nonlinearity.

Figure 5b illustrates the experiment configuration. A segment fiber of ~500 mm in length is, respectively, glued on a fixed and motorized stage. When the motorized stage moves from 0 mm to 1.5 mm, a strain ranged from 0 με to 3000 με is loaded on the fiber sensing. Figure 7 shows the displacement step is ~150 με and the wavelength shift verse the strain. The spatial resolution is 3 mm during the demodulation of OFDR. The laser sweep range is from 1545 nm to 1549 nm and the sweep rate is ~100 nm/s. The function of the fitting curve is Δλ(unit: pm) = 1.179 × ε(unit: με) + 0.8192. The experimental result indicates that the sensitivity is ~1.179 pm/με and the maximum fitting error is less than 20 με. Accordingly, the sensing nonlinearity in a full range of 3000 με is less than 0.67%.

### 3.3. Experiment on the Sensing Stability

**Figure 8:**
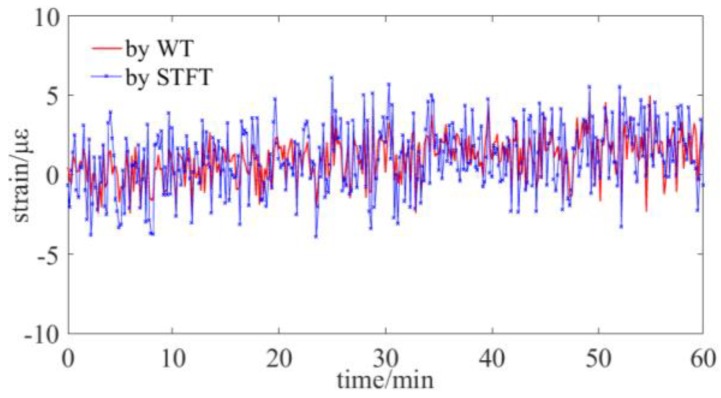
Experiment on the sensing stability of strain by WT and STFT.

Subsequently, the sensing stability of strain by the WT and STFT method is tested. The fiber sensing gauge is located at ~15 m and it is placed in a temperature controller box. There is no strain loaded on the fiber gauge during the experiment. The strain is sampled per 10 seconds and the experiment continues for 60 minutes. The experimental result is illustrated in Figure 8 and the peak-to-peak value, which indicates the sensing stability is ~7.3 με for WT and 12.1 με for STFT.

### 3.4. Experiment on the Sensing Resolution of Strain

**Figure 9:**
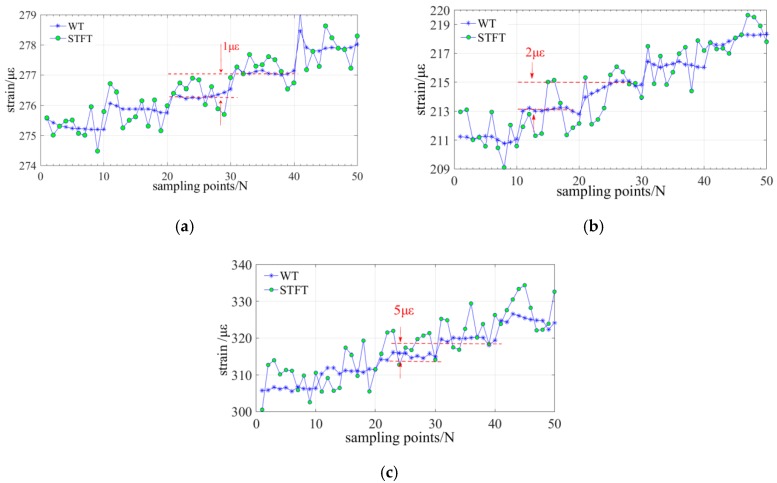
Experiment on the strain resolution under different spatial resolution: (**a**) spatial resolution is 20 mm; (**b**) spatial resolution is 8 mm; and, (**c**) spatial resolution is 5 mm.

The strain resolution is the key parameter of the distributed sensing and it is tested under different spatial resolutions of 20 mm, 8 mm, and 5 mm. The sampling time interval of every points is ~10 ms. The fiber sensing gauges is located at ~15 m and Figure 5b shows the experimental configuration. Figure 9a–c illustrates the experimental results and indicates that the strain resolutions under different spatial resolution of 20 mm, 8 mm, and 5 mm are, respectively, better than 1 με, 2 με, and 5 με. It also compares the strain resolution of WT with STFT method, and it indicates that the WT method could achieve a higher strain resolution and a lower noise under a highest spatial resolution of 5 mm.

### 3.5. Experiment on the Average Repeatability of the Distributed Strain

**Figure 10:**
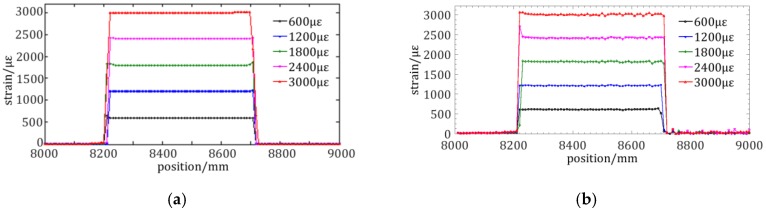

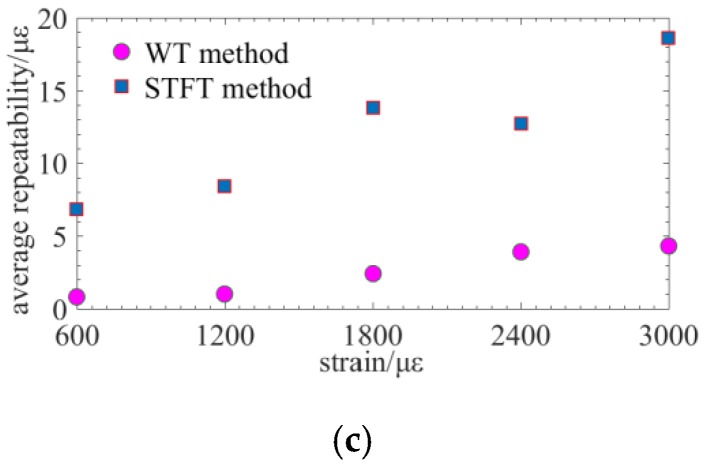
Experiment on the average repeatability of the distributed strain: (**a**) distributed strain achieved by the proposed method; (**b**) distributed strain achieved by the conventional STFT method; and, (**c**) the average repeatability of the distributed strain of the two methods.

A distributed strain of 600 με, 1200 με, 1800 με, 2400 με, and 3000 με is loaded on the fiber gauge of ~0.5 m length located between 8.2 m and 8.7 m. The distributed strain results that are achieved by the proposed WT method and STFT method are respectively illustrated in Figure 10a,b. The result of the proposed method is more stable in the region with distributed strain and the noise level is much lower in the region without any distributed strain. The performance of the proposed method can thus be verified.

Subsequently, the average repeatability of different strain is derived from Figure 10a,b. The repeatability is measured and it reflects 2*σ* standard deviation from the stable distributed sensing region. The average repeatability is the mean with sample size of 150 scans. The repeatability of 600 με, 1200 με, 1800 με, 2400 με, and 3000 με under a spatial resolution of 5 mm is less than 4.3 με, which is better than 18.6 με that is achieved by STFT method. The repeatability is improved by ~4.3 fold through using the proposed method. The result of a 5 mm gauge length is better than that of LUNA ODiSI-B (5.3 με), representing the leading level of OFDR.

### 3.6. Experiment on Sensing the Distributed Strain on the Surface of An Aluminum Alloy Plain

Finally, the proposed OFDR method is utilized to measure the distributed strain on the surface of an Aluminum alloy plain. The spatial resolution is 5 mm and the interval of fiber gauges is 3 mm. There are 40 measured points along one segment along axis *x* and there are 440 measured points on the surface.

**Figure 11:**
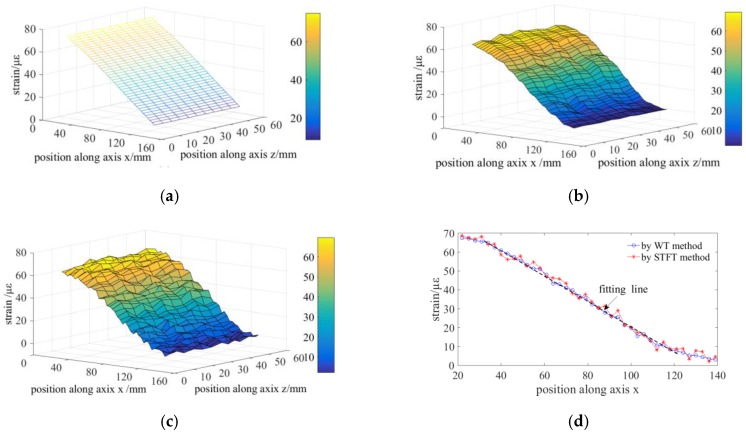
Experiment on sensing the distributed strain on the surface of an Aluminum alloy plain: (**a**) simulation result; (**b**) measured result by WT method; (**c**) measured result by STFT method; and, (**d**) comparison of the distributed strain curve along axis z located at 25 mm achieved by WT and STFT method.

A displacement of 1 mm is loaded at the free end of the cantilever Aluminum alloy plain. Figure 11a shows the simulation result by ANSYS. The strain *ε_x_* on the surface is linearly varying along axis *x* and its range is from 6.9 µε to 79.7 µε. Figure 11b,c illustrate the reconstructed strain on the surface measured by the proposed method and STFT method. The trend of the measured result is similar to the simulation result and the range of strain *ε_x_* is from 2.1 µε to 69.6 µε. The distributed strain measured by the fiber gauges is ~13% lower than the simulation result, which may be caused by the strain transmission efficiency of the glued fiber and position error during gluing fiber onto the surface. The proposed ST method could achieve a smooth distributed strain as a result of a low quantization error and the high spectrum resolution. Figure 11d indicates the distributed strain curve along axis z located at ~25 mm and the comparison result shows that the WT method could achieve a higher linearity than STFT method.

## 4. Conclusions

In this paper, a signal demodulation method that is based on WT is proposed to enhance the distributed sensing performance of an OFDR system. Its signal demodulation can be considered as a time-frequency analysis process based on the sensing principle of OFDR. A novel demodulation method based on WT is proposed to reduce the trade-off between spatial and spectrum resolution. The relationship between the distributed sensing parameters and wavelet parameters are demonstrated and several issues, such the parameters optimization, spatial varying with the location, and IF noise are solved. Finally, a signal processing tool that is based on Morlet WT is built to achieve OFDR distributed sensing. The performance of the proposed method is experimentally tested and compared with the STFT method in detail. The experimental results indicate that the spatial resolution can reach 5 mm, stability is less than ~7.3 με in peak-to-peak vale within one hour, the strain resolution is better than 1 με, the noise of the distributed strain achieved by the proposed method is reduced, and the average repeatability of the distributed strain is raised by ~4.3 fold in comparison to the STFT method. This method is promising for improving the resolution of both spatial and spectrum or measured distributed sensing physical quantity.
